# Antimicrobial and Physicochemical Properties of Artificial Saliva Formulations Supplemented with Core-Shell Magnetic Nanoparticles

**DOI:** 10.3390/ijms21061979

**Published:** 2020-03-13

**Authors:** Katarzyna Niemirowicz-Laskowska, Joanna Mystkowska, Dawid Łysik, Sylwia Chmielewska, Grażyna Tokajuk, Iwona Misztalewska-Turkowicz, Agnieszka Z. Wilczewska, Robert Bucki

**Affiliations:** 1 Department of Medical Microbiology and Nanobiomedical Engineering, Medical University of Bialystok, Mickiewicza 2C, 15-222 Bialystok, Poland; katia146@wp.pl (K.N.-L.); sylwia.chmielewska@umb.edu.pl (S.C.); buckirobert@gmail.com (R.B.); 2 Institute of Biomedical Engineering, Bialystok University of Technology, Wiejska 45C, 15-351 Bialystok, Poland; d.lysik@pb.edu.pl; 3 Department of Integrated Dentistry, Medical University of Bialystok, M. Sklodowskiej-Curie 24a, 15-276 Bialystok, Poland; grazyna.t1@gmail.com; 4 Faculty of Chemistry, University of Białystok, Ciolkowskiego 1K, 15-245 Bialystok, Poland; i.misztalewska@uwb.edu.pl (I.M.-T.); agawilczuwb@gmail.com (A.Z.W.); 5 Department of Microbiology and Immunology, The Faculty of Medicine and Health Sciences of the Jan Kochanowski University in Kielce, Stefana Żeromskiego 5, 25-001 Kielce, Poland

**Keywords:** saliva, magnetic nanoparticles, antimicrobial, biofilm, nanomedicine

## Abstract

Saliva plays a crucial role in oral cavity. In addition to its buffering and moisturizing properties, saliva fulfills many biofunctional requirements, including antibacterial activity that is essential to assure proper oral microbiota growth. Due to numerous extra- and intra-systemic factors, there are many disorders of its secretion, leading to oral dryness. Saliva substitutes used in such situations must meet many demands. This study was design to evaluate the effect of core-shell magnetic nanoparticles (MNPs) adding (gold-coated and aminosilane-coated nanoparticles NPs) on antimicrobial (microorganism adhesion, biofilm formation), rheological (viscosity, viscoelasticity) and physicochemical (pH, surface tension, conductivity) properties of three commercially available saliva formulations. Upon the addition of NPs (20 µg/mL), antibacterial activity of artificial saliva was found to increase against tested microorganisms by 20% to 50%. NPs, especially gold-coated ones, decrease the adhesion of Gram-positive and fungal cells by 65% and Gram-negative bacteria cells by 45%. Moreover, the addition of NPs strengthened the antimicrobial properties of tested artificial saliva, without influencing their rheological and physicochemical properties, which stay within the range characterizing the natural saliva collected from healthy subjects.

## 1. Introduction

Increasing the use of drugs against hypertension, diabetes, allergies, and depression [1] reflects an increasing number of people suffering from civilization diseases, and this is associated with reduced salivation causing oral dryness, known as xerostomia [2]. Hyposalivation is also recognized as a consequence of anticancer therapy, especially in the case of cancers that are located within the head and neck area [3]. 

Additionally, current progress in cancer treatment is associated with an increasing number of immunocompromised patients, characterized by a higher risk of opportunistic infections. Aggressive chemo- and radiotherapy cause inescapable oral toxicity, manifested by disruption of the mucosal barrier, leading to the development of inflammation in the oral cavity such as mucositis, gingivitis, periodontitis and leukoplakia [4]. In accordance with the literature [5] approximately 46–67% of BMS patients complain of dry mouth. Burning mouth syndrome (BMS) is defined as a burning sensation of the oral mucosa, in the absence of specific oral lesions. Peripheral alterations may be related to the density or reactive capacity of the oral mucosal membrane receptors. In the group of local causes, authors [6] distinguish biological factors that irritate mucosa directly (fungal, viral, bacterial infection) or mechanically (maladjusted dentures, poorly-fitting fillings, parafunctions). As a result, a variable number of these patients may suffer from lack of lubrication and become more prone to develop infections, because of reduced local host defenses.

Imbalance in the microbiota and oral infections caused by opportunistic pathogens were reported as well. As described by Panghal et al. [7], the list of microorganisms causing oral infections in a group of cancer patients includes *Staphylococcus aureus*, *Escherichia coli*, *Staphylococcus epidermidis*, *Pseudomonas aeruginosa*, *Candida spp,* and *Aspergillus spp.* The main reasons for an increase of pathogenic bacteria content possessing the ability to form biofilm are irradiation-induced histological changes within oral mucosa as well as changes in salivary glands function [8]. Indeed, the observed alterations in the microbiota of the oral cavity are mostly caused by a disturbance in the production of saliva [9], which plays a crucial role in the regulation of oral microbiota [10]. It is worth underlining that the growth of oral biofilm, e.g., on metal surfaces, can cause serious side effects, such as microbiologically influenced corrosion [11]. 

A recent study [12] indicates a direct relationship between the colonization of the oral cavity by Gram-negative *bacilli* and *Candida spp*. with the number of chemotherapy cycles and combined cancer therapy. Additionally, apart from the secretion function of salivary glands, it is established that radio- and chemotherapy might cause the reduction of phagocytic activity of salivary granulocytes [13]. In effect, the observed raising rate of oral complications in a group of patients subjected to cancer treatment makes mouth care a particular priority. Moreover, implementation of the prophylactic medication supporting the balance of oral microbiota should be incorporated along with cancer therapy.

In light of the above-listed problem, the development of artificial saliva preparations to reduce symptoms of dry mouth and to improve the quality of patients’ life have been proposed by several investigators [14,15,16,17]. To date, the use of different substitutes, which are based on carboxymethylcellulose, glycerol, and mucin, has been reported [18,19]. Moreover, they also contain different biological-active agents, such as natural plant extracts, herbs, and enzymes [20]. Nevertheless, in all cases, research efforts are also directed to develop artificial saliva with appropriate rheological, physicochemical and biological characteristics, to optimally mimic the natural saliva [21]. Those include lubricating, antimicrobial and immunomodulatory properties that are crucial in the protection of mucosal membrane, tissues, teeth, adequate functioning of the speech apparatus and food intake. However, to achieve ideal composition, which will fit all listed criteria of artificial saliva substitutes, more studies and novel solutions should be provided [14,22]. 

Recent progress in the application of nanotechnology has efficiently accelerated the development of different industries [23,24]. Among many types of available nanoparticles, metallics, especially iron-oxide nanoparticles (NPs), have attracted more attention, due to their unique physicochemical properties and biocompatibility [25]. They are promising candidates in the biomedical application, including carriers in drug delivery systems, MRI-contrast agents, hyperthermia inducers and photosensitizers in the diagnostic and therapeutic areas [26,27]. Moreover, several studies proved their potential as antimicrobial agents that prevent biofilm formation [28,29]. To date, among metallics’ nanomaterials, usage in the area of dentistry silver and gold particles has been successfully engaged [30,31,32]. To the best of our knowledge, this is the first study where core-shell magnetic nanoparticles were proposed and tested as a component of artificial saliva preparation to improve their antimicrobial activity. The influence of NPs on physicochemical and rheological properties such as pH, conductivity, surface tension, viscosity and viscoelasticity of saliva substitutes were assessed as well. 

## 2. Results and Discussion

**Figure 1:**
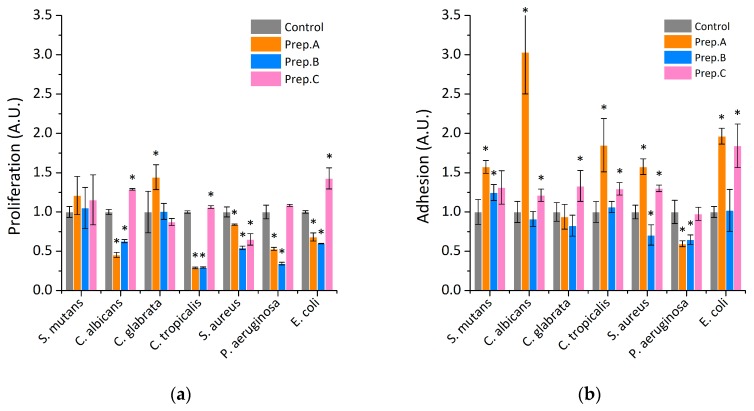

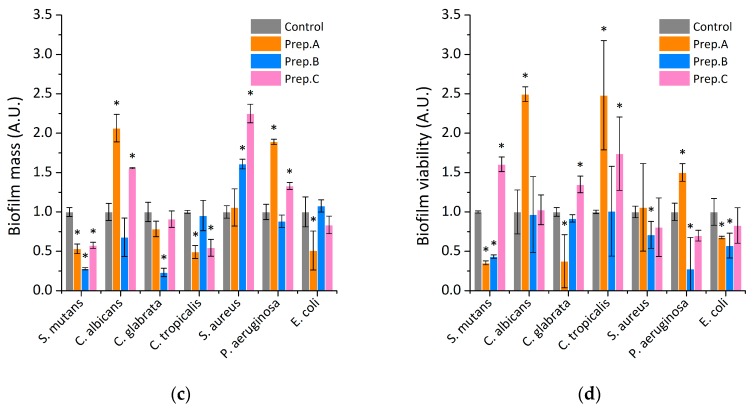
The effect of tested artificial saliva preparations on the growth of representative oral microorganism. Panel (**a**) indicate the ability of tested preparations to restrict their division. Panel (**b**,**c**) shows the influence of tested saliva preparations on the initial stage (adhesion) and the formation of biofilm. Viability of the cell embedded in the biofilm matrix after treatment by tested saliva formulations are presented at panel (**d**). Statistical significance for the tested preparations compared to control was marked by (*) *p* ≤ 0.05. The data showed medium results from three measurements ±SD.

In the first step of biological tests, we evaluated the ability of verified, commercially available preparations of artificial saliva to inhibit the proliferation of selected oral pathogens (Figure 1a). It is important to underline that the multiplication of microorganisms is an important step that allows colonization. For this purpose, bacterial or fungal pathogens were incubated in growth medium in the presence of tested substitutes for 6 h. Results showed, that only in the case of substitute B, restriction of microorganism grow has been noted. The best effect was observed in the case of *P. aeruginosa* and *C. tropicalis*, where proliferation was classified at the level of 30% in comparison to control. The 50% inhibition was noted after incubation of evaluated substitute B with *S. aureus*, *E. coli,* and *C. albicans*. In other tested conditions, it was indicated that the addition of preparation A has a lower ability to restrict the proliferation of the oral pathogens, while in the case of preparation C, a lack of impact on cell division process has been noted. Importantly, in the case of *S. mutans*, which are known as crucial bacterial pathogens that are responsible for dental plaque formation, it was noted that the addition of all tested substitutes has no influence on the microorganisms‘ proliferation process. In turn, the evaluation of anti-adhesion properties of tested substitutes indicated that, among them, the best protective effect was noted for preparation B (Figure 1b), where, in comparison to control, 10% to 20% depletion of microorganism adherence has been observed. However, in the rest of the cases, lack of influence or increasing rate of the adhesion process was noted. This provides evidence that commercially available artificial saliva preparation does not adequately protect against the initial stage of dental plaque formation. In the next step, the preventive activity of evaluated substitutes against the formation of biofilm has been assessed (Figure 1c). Substitute B showed the best inhibitory properties against the formation of biofilm. Importantly, in the case of *S. mutans*, 75% and 50% reduction of the biofilm mass have been observed, after being cultured in the presence of substitute B and substitute A and C, respectively. Similarly, in the case of the impact of assessed preparations on the viability of the cell, embedded on the biofilm matrix, some restriction activity was observed only for preparation B (Figure 1d), for all tested oral pathogens.

As previously mentioned, the tested saliva preparations contain components that exhibit antimicrobial properties. Some of these ingredients (e.g., hydroxyethylcellulose, Yerba Santa extract) might form the natural protective layer on the mucosal surfaces, preventing microbial colonization. Other components (e.g., xylitol, sorbitol, system of enzymes, fluoride ions) have antimicrobial properties, prevent growing of harmful flora. Results of this study show that preparation B, based on xylitol, hydroxyethylcellulose and system of enzymes, exhibits the best antimicrobial activity, confirming previous reports [33,34], indicating that those enzymes were successfully used as main antimicrobial agents.

**Figure 2:**
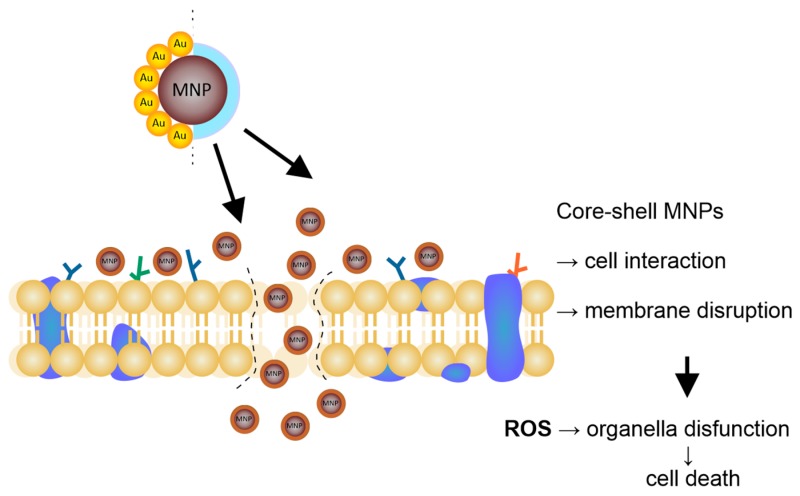
The mechanism of nanoparticles’ activity within the cell membrane and cytosol.

However, to increase the activity of tested formulations, and to prevent microbial adhesion, processes that are required to achieve colonization and formation of dental plaque, and modification of the preparation content by the addition of core-shell magnetic nanoparticles was conducted (working concentration of 20 g/mL). Proposed nanostructures possess well-defined physicochemical properties and proven antibacterial activity. According to published literature [35,36,37], the proposed mode of antibacterial action of magnetic nanoparticles involves their contribution in the induction of oxidative stress, via generation of reactive oxygen species (ROS), as well as interference with the bacterial electron transport of the oxidation of NADH. Moreover, they possess the ability to disrupt the membrane of microorganisms via the formation of a pore or by the endocytosis based mechanism shown in Figure 2.

**Figure 3:**
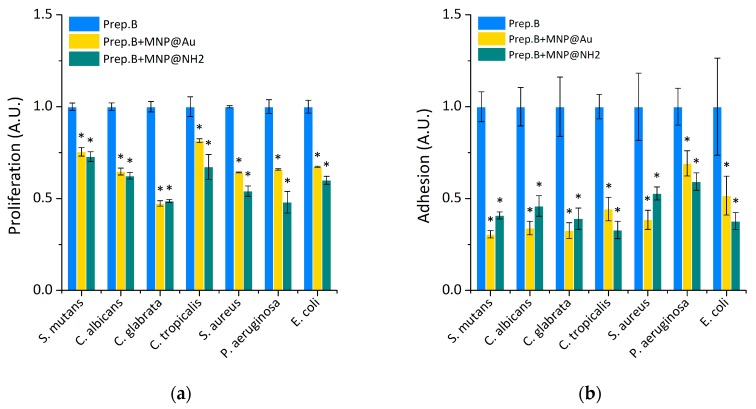

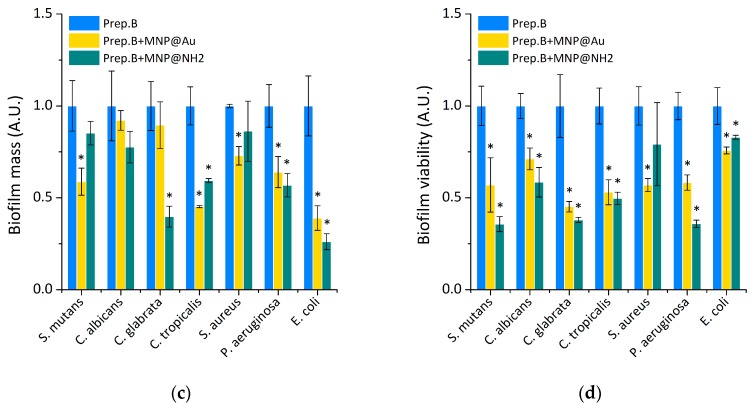
The addition of magnetic nanoparticles to artificial saliva preparations inhibits the growth and biofilm formation of selected oral pathogens. Panel (**a**) indicated the ability of tested formulations to restrict the proliferation of selected oral pathogens. Panels (**b**,**c**) show the ability of tested saliva preparations containing magnetic nanoparticles against the microorganism adhesion and the formation of biofilm. Decreased viability of the cells embedded in the biofilm matrix after treatment with tested saliva formulations containing magnetic nanoparticles is presented at panel (**d**). Statistical significance for the tested preparations, compared to control, was marked by (*) *p* ≤ 0.05. Results from three measurements ±SD.

Our previous studies [38,39,40,41] have shown that they interact in a synergistic or additive manner, with chemotherapeutic agents used in standard antimicrobial treatment, as well as with a representative of antimicrobial peptides that take part in the immune-defense process and with their synthetic mimetics. Here we describe how the presence of magnetic nanoparticles might lead to improving the antimicrobial properties of artificial saliva substitutes (Figure 3). In brief, in the case of the ability to restrict the proliferation process, the addition of core-shell magnetic nanoparticles provide better efficacy of tested preparation, which is classified at being in the range between 20% to 50%, in comparison to preparation B alone (Figure 3a). Moreover, we observed that the presence of nanoparticles dramatically decreases the adhesion process—on average 65% (for Gram-positive and fungal cells) and ~45% (for a representative of Gram-negative bacteria cells) lower affinity of the evaluated pathogens to the tested surface was noted (Figure 3b). At the tested conditions, there was a mean reduction of biofilm mass of ~70% in the case of *E. coli*, and ~40% in the cases of *P. aeruginosa* and *C. albicans*, regardless of the kind of nanoparticles (Figure 3c). However, in the case of Gram-positive bacteria, significantly better activity was noted after the addition of gold-coated nanoparticles. In turn, in the aspect of viability of cells embedded in biofilm mass, addition of tested substitutes supplemented by magnetic nanoparticles indicated that means reduction of viable cell was calculated at the level 55 % in the case of fungal cells, 40% in the case of Gram-positive bacteria and 50 % for *P. aeruginosa* (Figure 3d).

**Figure 4:**
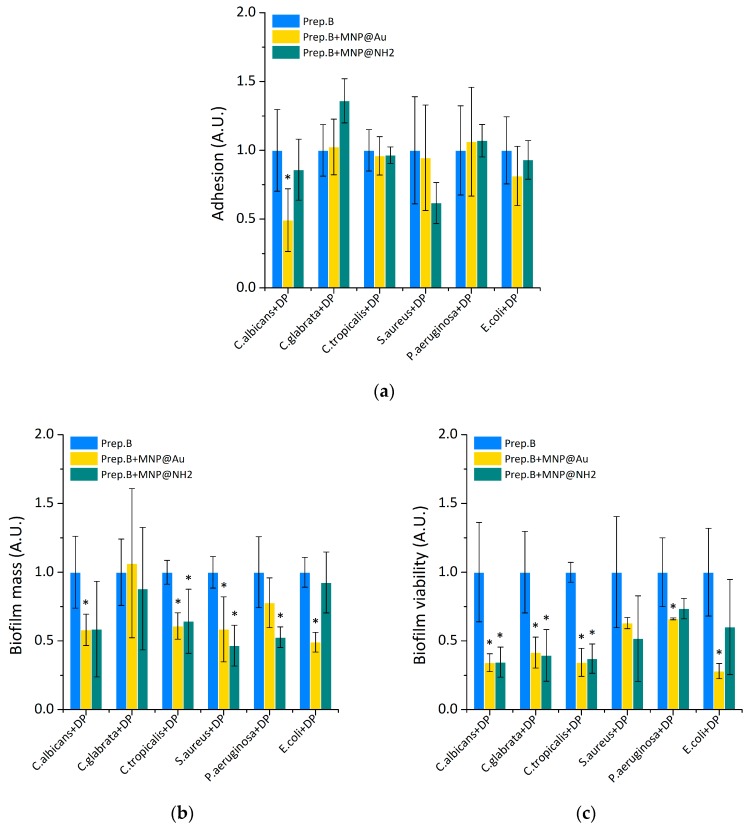
The addition of magnetic nanoparticles to artificial saliva preparations inhibits the growth and biofilm formation of selected oral pathogens in the presence of dental plaque (DP). Panels (**a**,**b**) show the ability of tested saliva preparations containing magnetic nanoparticles against the microorganism adhesion and the formation of biofilm in the presence of dental plaque. Decreased viability of the cells embedded in the biofilm matrix after treatment with tested saliva formulations containing magnetic nanoparticles in the presence of dental plaque is presented at the panel (**c**). Statistical significance for the tested preparations compared to control was marked by (*) *p* ≤ 0.05. Results from 3 measurements ±SD.

**Figure 5:**
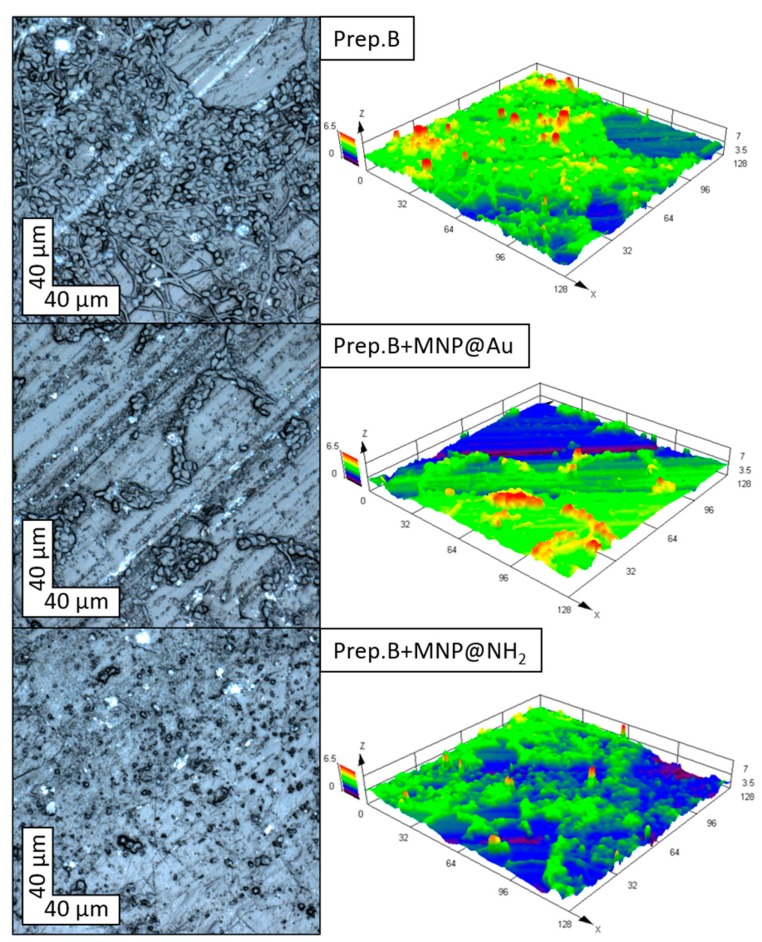
CLSM images of *C. tropicalis* 48 h biofilm treated with preparation B and preparation B with the addition of magnetic nanoparticles MNP@Au and MNP@NH_2_.

In physiological conditions, saliva plays a major role in determining the nature and activity of the oral microbiota. For this purpose, different mechanisms are engaged. Saliva controls the pH around neutrality, in effect creating an environment that is adequate for the growth of many oral microorganisms, and provides a relationship developing between the host and the resident microbiota. However, any perturbation in the flow of saliva, including xerostomia, might cause dysbiosis to occur rapidly [14]. Oral cavity bacteria, specifically those involved in biofilm formation and dental disorders, are major health concerns. Recently published results show the low impact of commercially available artificial saliva preparations on the restriction of microorganism adhesion in the presence of the representatives of oral bacteria—*S. mutans*. Thus, in this work, the ability of preparation B containing gold- or aminosilane coated magnetic nanoparticles was assessed in vitro, for inhibition of the adhesion and biofilm formation of tested microorganisms co-culturable with oral bacteria (Figure 4). As published previously [14], the evaluation of the ability of artificial saliva preparations to restrict the adhesion process indicated that, in most cases, only substitute B possesses the ability to significantly decrease the attachment of tested microorganisms to the surface. The addition of nanoparticles does not decrease or slightly decrease the adhesion efficacy. Only in the case of *C.albicans*, significant depletion of adhesion (more than 50% in comparison to preparation B) has been observed for gold-coated nanoparticles. Importantly, both the NPs, at a concentration of 20 μg/mL, significantly inhibited the formation and viability of microorganisms’ biofilm in the presence of oral bacteria, if compared to preparation without magnetic nanoparticles. The results of microscopic observations of 48 h *C. tropicalis* biofilm shown in Figure 5 confirm the reduction of biofilm growth in the presence of preparation B, with the addition of MNP@Au or MNP@NH2 in comparison to unmodified saliva substitute B.

**Figure 6:**
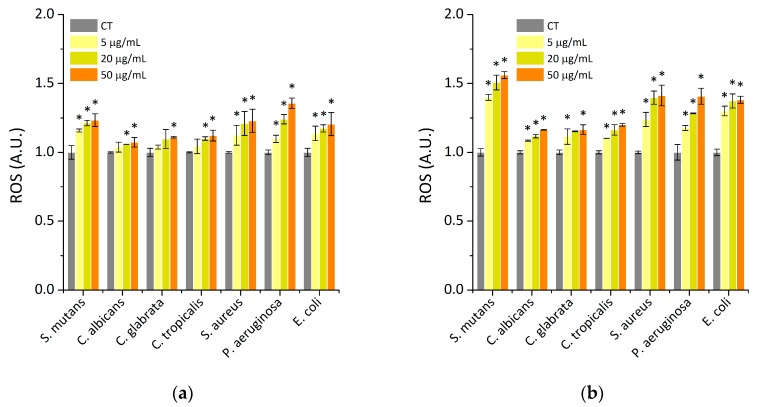
Results of reactive oxygen species (ROS) generation: (**a**) MNP@Au; (**b**) MNP@NH_2_. Statistical significance for the tested preparations compared to control was marked by (*) *p* ≤ 0.05. Results from three measurements ±SD.

It is established that three major mechanisms of nanomaterials are responsible for their antimicrobial properties, including membrane damage and cellular uptake; releasing toxic metals, which can react with macromolecules, causing a loss of their function, and in effect restricting grow or killing microbes; and generating reactive oxygen species (ROS), which cause DNA, RNA, and proteins damage [42]. Many recent studies indicated that magnetic nanoparticles can produce reactive oxygen species (ROS), which play a major role in various cellular pathways, via the Fenton and Haber–Weiss reaction [43]. It is established that an increased level of ROS production can cause the oxidation of protein and nucleic acids, which in effect leads to cellular damage. To study the effect of saliva- containing nanosystems on the generation of oxidative damage, ROS production has been measured (Figure 6). For this purpose, the fluorescence-based technique was employed. It was indicated that the higher potential of oxidative damage of the treated microorganisms was observed in the case of aminosilane coated MNP, rather than in the case of gold-coated MNPs. However, in the gold-coated MNPs, the ability to generate ROS is dependent on the type of treated pathogens; for example for bacteria cells, the oxidative damage was observed after addition of NPs at lowest concentration (5 µg/mL), while in the case of fungi concentration, between 20 to 50 μg/mL is required. A similar observation was detected in the case of aminosilane coated MNPs, however, the ROS generation was observed after the addition of MNPs at all tested concentration ranges and in most cases, the effect depended on the applied concentration of MNPs. Obtained results have shown that nanosystems proposed in this study caused oxidative damage in bacterial and fungal cells, but at a slight level. This is in agreement with previously published studies that indicated that the coated systems displayed negligible ROS generation compared to uncoated nanoparticles. However, this is one part of their mode of action which determines their antimicrobial effect [42]. Our previously published studies have demonstrated that they strongly interact with cell-wall molecules, including specific protein such as protein F [39,44]. The above mentioned mode of MNPs action is associated with the ability to disrupt the plasma membrane of microorganisms. Moreover, recently it has been published that the application of magnetic nanoparticle/alternating magnetic field (MNP/AMF) hyperthermia could promote the bactericidal activity of macrophages against intracellular bacteria, via the MNP-dependent generation of reactive oxygen species [45]. This suggests the immunomodulatory role of the nanoparticles and provides the opportunity for the application of our preparations in the case of immunocompromise and/or cancer-patients as an adjuvant method of therapy.

**Figure 7:**
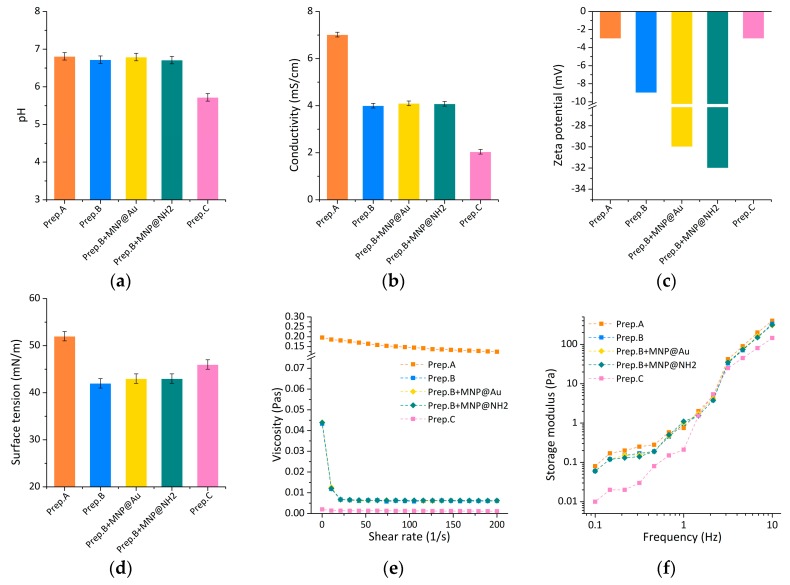
Physicochemical and rheological characteristics of tested artificial saliva preparations: (**a**) pH, (**b**) conductivity, (**c**) zeta potential, (**d**) surface tension, (**e**) viscosity, and (**f**) viscoelasticity. Shown data refer to average-values of five measurements.

Taking into account that those saliva preparations play a different role in the oral cavity, their complex properties are important from a practical point of view. In the aspect of the dental practice, saliva is also responsible for the proper use of prosthetics and orthodontics elements and dental fillings, especially in the field of neutral pH and lubrication ability. Thus, in the second part of the work, the physicochemical and rheological properties were evaluated. As shown in Figure 7a, the lowest pH (~5.6) is observed for preparation C. The inorganic fraction ions act as a buffer to maintain the pH of the saliva between six and seven. It should be emphasized here that a saliva pH value that is too low is unfavorable when considering the prevention of tooth demineralization and the corrosion processes of metal elements used in dental applications [11,46]. However, the buffering mechanism can be limited by a high density of microbial cells or by a low salivary flow rate [47]. In the case of other substitutes, both unmodified and modified by nanoparticles, pH is in the range of 6.8–6.9, which is similar to the pH of natural human saliva, which was presented in our previous work [48]. In the case of conductivity tests (Figure 7b), the highest value (~8.5 mS/cm) was observed in the case of preparation A and the lowest (~2 mS/cm) for preparation C and simultaneously, according to literature [48], this value was the most similar to the conductivity of human saliva (~1.4 mS/cm). In the composition of saliva substitute C are organic biomolecules with long chains, which decrease its conductivity. For all B preparations, conductivity was in the range of 4.0–4.1 mS/cm, with a little higher value for nanoparticles-based saliva substitutes. This physicochemical parameter influences ions transport in the oral cavity, e.g., fluoride, which may be beneficial for anti-caries protection.

The stability of the saliva and saliva modified by MNP@NH_2_ or MNP@Au nanoparticles was measured. As is shown in Figure 7c, zeta potentials for tested preparations are adequately: A (−2.4 mV), B (−9.0 mV) and C (−2.5 mV). In the case of preparation B, modified by the above additives, the stabilizing effect of nanoparticles was observed. The change of zeta potential from −9 mV to −32 mV for substitute B modified with MNP@NH_2_, and to the value of −30 mV for the substitute B modified with MNP@Au, was obtained. Simultaneously, some increase in particle size was observed—the addition of nanoparticles caused the formation of some aggregates (1400 nm for preparation B with MNP@NH_2_ and 750 nm for preparation B with MNP@Au). However, it does not cause the destabilization of saliva preparation, as a high value of zeta potential was preserved. The aggregation can be explained by the formation of new hydrogen bonds between saliva and MNP@NH_2_ or MNP@Au nanoparticles. The samples still possess free –OH groups that come from the magnetic core. Apart from the formation of some aggregates, the discussed samples are stable.

The next of the tested physicochemical parameters, surface tension, in addition to rheological properties, are responsible for lubrication in the oral cavity. The surface tension of natural saliva was reported [48,49] to range between 47–58 mN/m. As Figure 7d showed, the highest value of this parameter was observed for substitute A (~53 mN/m), the medium value was obtained for substitute C (~46 mN/m) and the lowest for substitute B (~42–43 mN/m). From the lubrication point of view, better results are achieved in cases of low surface tension, as in this case, as the wettability of the oral cavity surface is higher. In this study, all tested saliva substitutes before and after nanoparticles’ modification are characterized by lower surface tension than natural saliva. It can decrease the friction between surfaces in the oral cavity and improve the retention of removable dentures.

The role of saliva in the oral health system is important. In addition to biological functions or related to maintaining homeostasis in the oral cavity, it is also responsible for reducing friction between surfaces in contact [50], due to its viscous and viscoelastic properties [51,52]. In particular, these are opposing teeth, dental fillings, orthodontic and prosthetic components. Saliva reduces the resistance of movement between moving elements relative to each other, and thus, reduces friction, resulting in reduced wear.

The shearing curves of tested solutions at different shear rates, between 0.01 1/s to 200 1/s, are presented in Figure 7e. The tested preparations were characterized by different viscosities, from 0.001 Pas (for preparation C) to 0.20 Pas (for preparation A), at a shear rate of about 20 1/s. The lowest value was observed for substitute C and was stable during tests. For preparations B and C, as the shear rate increased, the dynamic viscosity decreased. It was especially observed in the case of preparation A, which proves its pseudoplastic properties. An increase of shear rate may be destructive to organic aggregates of substitute A (e.g., xylitol, poloxamer, hydroxyethylcellulose), resulting in a decrease of its viscosity. However, the viscosity of substitute B (~0.007 Pas) was the most similar to the viscosity of natural saliva (~0.01 Pas), which was tested in work [21]. Obtained results show no influence of core-shell magnetic nanoparticles on artificial saliva viscosity.

The second stage of rheological tests involved viscoelasticity tests, with the goal to assess the elastic modulus. This storage module provides information on the conditions in which the molecular structure of biomolecules remains unchanged, due to the possibility of stress absorption. On the other hand, it represents the ability of a substance to accumulate energy during deformation. For natural saliva, this module is 3.5 Pa at 1 Hz [52]. At work, tests were performed for a frequency sweep in the range of 0.1–10 Hz, and strain amplitude equal to 0.1. As shown in Figure 7f, a higher storage modulus (~1 Pa at 1 Hz) was observed for preparations A and B, based on xylitol, poloxamer, hydroxyethylcellulose and xylitol, glycerine, cellulose gum adequately. The elastic modulus of preparation C, based on sorbitol, xylitol and Yerba Santa extract, were lower (~0.11 Pa at 1 Hz), in comparison to preparations A and B. Obtained results show that the addition of core-shell magnetic nanoparticles has no influence on the viscoelastic properties of tested artificial saliva. 

In summary, it should be emphasized that the addition of NPs strengthened the antimicrobial properties of tested artificial saliva preparations. However, their physicochemical and rheological parameters relevant to the functional activity have been preserved.

## 3. Materials and Methods

### 3.1. Synthesis and Characterization of Core-Shell Magnetic Nanoparticles

**Figure 8:**
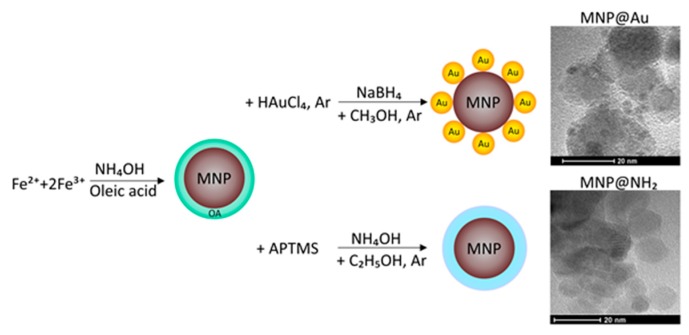
Synthesis and morphology of magnetic nanoparticles coated by gold (MNP@Au), and with a terminal amine group (MNP@NH_2_) attached to their surface.

Magnetic nanoparticles with gold- or aminosilane shells were obtained as described previously, with some modification [38]. In brief, the iron-oxides core was synthesized via modification of Massart’s method, which was based on the co-precipitation procedure of iron chloride salts in base conditions. Gold functionalized nanoparticles were synthesized by using the modification of the reduction procedure. Aminosilane shell was obtained by one-step polycondensation of (3-aminopropyl)trimethoxysilane (APTMS). The synthesis and morphology of core-shell magnetic nanoparticles are shown in Figure 8. After that, in both procedures, the nanoparticles were washed three times with water and ethanol and allowed to dry in an oven at 50 °C overnight [53]. 

Core-shell nanoparticles were characterized using the following physicochemical methods, including FT-IR spectroscopy (Nicolet 6700 FT-IR spectrophotometer, Thermo Fisher Scientific, Waltham, MA, USA), differential scanning calorimetry (DSC Discovery apparatus, TA Instruments, New Castle, DE, USA) and thermogravimetric analysis (TGA Q500 thermogravimetric analyzer, TA Instruments, New Castle, DE, USA), dynamic light scattering and zeta potential analysis (Zetasizer Nano ZS, Malvern Instruments, Malvern, UK), and transmission electron microscopy TEM/EDX (Tecnai G2 X-TWIN, FEI Company, Hillsboro, OR, USA). Data are already published [39]. 

### 3.2. Artificial Saliva Preparations

Three commercial saliva substitutes were tested. All preparations, according to the manufacturer’s information, are characterized by moisturizing and soothing dryness, as well as antimicrobial and refreshing properties. Next to inorganic compounds affecting physicochemical properties, in each preparation, there are organic active compounds responsible mainly for lubricating and antimicrobial properties. Especially in composition of substitute A, there are xylitol, poloxamer, hydroxyethylcellulose, and a system of three enzymes; in the composition of substitute B there are xylitol, glycerine, and cellulose gum; in the composition of substitute C there are sorbitol, xylitol and Yerba Santa extract. 

### 3.3. Proliferation Assay

To evaluate the effect of tested artificial saliva preparations on the growth of oral pathogens, cells were suspended to an optical density at 600 nm of 0.1 in LB or BHI growth medium. Then, the cells (100 μL) were placed into 96-well microtiter plates containing tested preparations (100 µL) and resazurin was added at a final concentration of 5 µg/mL. Fluorescence was recorded using Varioskan LUX multimode microplate reader (Thermo Fisher Scientific, Waltham, MA, USA) for 6 h at 37 °C, with excitation/emission wavelengths of 520/590 nm.

### 3.4. Adhesion Assay

To determine the anti-adhesion activity of tested artificial saliva preparations with or without core-shell magnetic nanoparticles, the CV-staining method was used. We compared the abilities of tested strains to adhere to wells of polystyrene microtiter plates in the presence of tested artificial saliva preparations. In brief, pathogen cells were suspended to an optical density at 600 nm of 0.1. In the next step, the cells (100 μL) were placed into 96-well microtiter plates containing tested preparations (100 µL) and incubated for 2 h at 37 °C. Then, 1% crystal violet (CV) (25 μL) was added to each well, and the plates were incubated at room temperature for 15 min. After that, each well was carefully rinsed with water, and ethanol (200 μL) was added to extract the CV. Optical density (OD) was measured at a wavelength of 580 nm, using a Varioskan LUX multimode microplate reader (Thermo Fisher Scientific, Waltham, MA, USA).

### 3.5. Biofilm Assay

To assess the ability of the tested preparations to restrict the formation of biofilm, representative of oral pathogens were grown for 48 h at 37 °C in the presence of artificial saliva. After incubation, each well was washed with PBS to eliminate unbounded planktonic cells. Biofilm mass was assessed using the crystal violet CV-staining (0.1%) method. The unbound stain was rinsed off with deionized water, and then ethanol (70%, 100 μL) was added and the optical density (OD) was measured at 580 nm using a Varioskan LUX multimode microplate reader (Thermo Fisher Scientific, Waltham, MA, USA).

In other sets of the experiment, to evaluate the viability of cells embedded in biofilm matrix during the formation of biofilm in the presence of tested preparations, MTT test, which was based on the reduction of tetrazolium salt to unsoluble formazan, was performed. Biofilm was formed over the course of 48 h, then unbonded planktonic cells were carefully rinsed using PBS. In the next step, after 3 h of incubation, dye was resolved in DMSO and the plates were spectrophotometrically measured at a wavelength of 540 nm, using a Varioskan LUX multimode microplate reader (Thermo Fisher Scientific, Waltham, MA, USA).

To determine the ability of the tested artificial saliva preparations to restrict adhesion and formation of the biofilm in physiological conditions, measurements were performed in the presence of 50% human dental plaque containing *Streptoccocus mutans*. Dental plaque was obtained from volunteers/patients. The trial was approved by the Human Research Ethics Committee of the Medical University of Bialystok, Poland (R-I-002/4/2019, 31 January 2019). For both studies, all subjects provided informed written consent and collected samples were anonymous.

### 3.6. Confocal Laser Scanning Microscopy (CLSM) Observations

Surfaces of the polymer samples with *Candida tropicalis* 48 h biofilm were observed using confocal laser scanning microscopy (CLSM) LEXT OLS 4000 (Olympus, Tokyo, Japan). Samples were observed just after being rinsed three times in pure water to remove free molecules from the polymer surface. Then, 2D surface reconstruction with biofilm was obtained in automatic mode. To obtain three-dimensional reconstructions of the biofilm structure, a sample scan was performed at a frequency of 0.05 micrometers in the Z-axis.

### 3.7. ROS Generation Assessment

The assessment of ROS generation by MNP@Au and MNP@NH_2_ nanoparticles in the presence of the following strains: *Staphylococcus aureus, Streptococcus mutans, Pseudomonas aeruginosa, Escherichia coli, Candida glabrata, Candida tropicalis, and Candida albicans*, was carried out, measuring the latter strains using 2′,7′-dichlorofluorescin diacetate (DFCH-DA) as a fluorescent probe. In the experiment, bacterial cells (OD_600_ = 0.1) and fungal cells (OD_600_ = 0.7) were pipetted into 96-well black plates. Then, nanoparticles (MNP@Au, MNP@NH_2_) at concentrations 5, 20 and 50µg/mL were added to each well in the presence of artificial saliva. After that, the solution of 20 µM DFCH-DA in PBS was prepared. Finally, fluorescence was measured for 60 min, immediately after the addition of the dye at excitation/emission wavelengths of 488/535 nm.

### 3.8. Physicochemical and Rheological Characterization of Artificial Saliva Preparations 

The SevenMulti (Mettler Toledo, Columbus, OH, USA) multifunctional ionoconductometr with special electrodes was used for measuring pH and conductivity. Tests of surface tension were conducted with a platinum ring using the STA1 tensiometer (Sinterface, Berlin, Germany). Both tests were performed at room temperature (21 ± 1 °C). The stability of saliva-MNP was studied using a Zetasizer Nano-ZS (Malvern Instruments, UK) equipped with a 4 mW helium/neon laser (l = 633 nm) and a thermoelectric temperature controller. All measurements were performed at 25 °C with a backscatter detection system at 173°. Rheological tests were carried out using the HAAKE Rheostress 6000 rheometer, with an automatic temperature module recognition Peltier system (Thermo Fisher Scientific, Waltham, MA, USA). Viscosity measurements of saliva preparations were performed in a cone-plate arrangement, where the diameter of the cone was 35 mm, and the angle between the slant height and the cone radius was 1 degree. The shear rate range in viscosity measurements was 0.1–200 1/s. The viscoelastic properties of the preparations were examined by the oscillation method using a system of two parallel plates, in which the diameter of the upper plate was 35 mm. Using the control strain mode, in the first step, the deformation range, in which the viscoelasticity modules are constant, was determined. On this basis, a frequency sweep in the range 0.1–10 Hz was carried out for the determined strain amplitude equal to 0.1. Rheological tests were carried out at 37 °C, where the maximum allowable temperature deviation was ±0.1 °C.

## 4. Conclusions

Our results showed that the addition of NPs modulates the antibacterial and antifungal activity of saliva substitutes by decreasing microorganism adhesion and biofilm formation. Additionally, nanoparticles restrict the proliferation of bacterial planktonic cells, as well as the viability of cells embedded in the biofilm matrix. Moreover, the physicochemical and rheological properties of tested saliva preparations with the addition of nanoparticles were not changed, indicating that nanoparticles will likely not interfere with the biophysical homeostasis of the oral cavity.
